# Adrenomedullin for steroid-resistant ulcerative colitis: a randomized, double-blind, placebo-controlled phase-2a clinical trial

**DOI:** 10.1007/s00535-020-01741-4

**Published:** 2020-11-02

**Authors:** Toshihiro Kita, Sinya Ashizuka, Naoki Ohmiya, Takayuki Yamamoto, Takanori Kanai, Satoshi Motoya, Fumihito Hirai, Hiroshi Nakase, Tomohiko Moriyama, Masanao Nakamura, Yasuo Suzuki, Shuji Kanmura, Taku Kobayashi, Hidehisa Ohi, Ryoichi Nozaki, Keiichi Mitsuyama, Shojiro Yamamoto, Haruhiko Inatsu, Koji Watanabe, Toshifumi Hibi, Kazuo Kitamura

**Affiliations:** 1 Division of Circulatory and Body Fluid Regulation, Department of Internal Medicine, Faculty of Medicine, University of Miyazaki, 5200 Kihara, Miyazaki, Miyazaki 889-1692 Japan; 2 Department of Gastroenterology, Fujita Health University School of Medicine, Toyoake, Japan; 3 IBD Center, Yokkaichi Hazu Medical Center, Yokkaichi, Japan; 4 Division of Gastroenterology and Hepatology, Department of Internal Medicine, Keio University School of Medicine, Tokyo, Japan; 5 IBD Center, Sapporo Kosei General Hospital, Sapporo, Japan; 6 Department of Gastroenterology and Medicine, Faculty of Medicine, Fukuoka University, Fukuoka, Japan; 7 Department of Gastroenterology and Hepatology, Sapporo Medical University School of Medicine, Sapporo, Japan; 8 Department of Medicine and Clinical Science, Graduate School of Medical Sciences, Kyushu University, Fukuoka, Japan; 9 Department of Gastroenterology and Hepatology, Nagoya University Graduate School of Medicine, Nagoya, Japan; 10 Department of Gastroenterology, Department of Internal Medicine, Toho University Sakura Medical Center, Sakura, Japan; 11 Digestive and Lifestyle Diseases, Kagoshima University Graduate School of Medical and Dental Sciences, Kagoshima, Japan; 12 Center for Advanced IBD Research and Treatment, Kitasato University Kitasato Institute Hospital, Tokyo, Japan; 13 Department of Gastroenterology, Idzuro Imamura Hospital, Kagoshima, Japan; 14 Coloproctology Center Takano Hospital, Kumamoto, Japan; 15 Division of Gastroenterology, Department of Medicine, Kurume University School of Medicine, Kurume, Japan; 16 Department of Gastroenterology and Hematology, Faculty of Medicine, University of Miyazaki, Miyazaki, Japan; 17 Department of Gastroenterology, Fukuoka University Chikushi Hospital, Fukuoka, Japan

**Keywords:** Adrenomedullin, Ulcerative colitis, Phase 2a clinical trial, Japanese

## Abstract

**Background:**

Adrenomedullin (AM) is a bioactive peptide having many pleiotropic effects, including mucosal healing and immunomodulation. AM has shown beneficial effects in rodent models and in preliminary study for patients with ulcerative colitis (UC). We performed a clinical trial to investigate the efficacy and safety of AM in patients with UC.

**Methods:**

This was a multi-center, double-blind, placebo-controlled phase-2a trial evaluating 28 patients in Japan with steroid-resistant UC. Patients were randomly assigned to four groups and given an infusion of 5, 10, 15 ng/kg/min of AM or placebo for 8 h per day for 14 days. The primary endpoint was the change in Mayo scores at 2 weeks. Main secondary endpoints included the change in Mayo scores and the rate of clinical remission at 8 weeks, defined as a Mayo score 0.

**Results:**

No differences in the primary or secondary endpoints were observed among the four groups at 2 weeks. Despite the insufficient tracking rate, the Mayo score at 8 weeks was only significantly decreased in the high-dose AM group (15 ng/kg/min) compared with the placebo group (− 9.3 ± 1.2 vs. − 3.0 ± 2.8, *P* = 0.035), with its rate of clinical remission at 8 weeks being significantly higher (3/3, 100% vs. 0/2, 0%, *P* = 0.025). We noted mild but no serious adverse events caused by the vasodilatory effect of AM.

**Conclusions:**

In this double-blind randomized trial, we observed the complete remission at 8 weeks in patients with steroid-resistant UC receiving a high dose of AM.

**Clinical trial registry:**

JAPIC clinical trials information; Japic CTI-205255 (200410115290). https://www.clinicaltrials.jp/cti-user/trial/Search.jsp.

**Electronic supplementary material:**

The online version of this article (10.1007/s00535-020-01741-4) contains supplementary material, which is available to authorized users.

## Introduction

Ulcerative colitis (UC) is an intractable disease that causes chronic inflammation or ulcers in the mucosa of the colon. In recent decades, many new medical regimens, such as biologics and immunosuppressants, have been introduced for the treatment of UC, with the clinical outcomes of patients being improved. Especially, biologics including antitumor necrosis factor (TNF) agents and anti-integrin antibodies have shown significant effectiveness even in intractable cases, such as steroid-refractory and steroid-dependent UC [1–3]. However, intractable cases in which mucosal healing has been disturbed remain untreatable even when using advanced anti-inflammatory therapies. So novel drugs contributing to the mucosal regeneration through new mechanisms are desired.

Adrenomedullin (AM) is a potent vasodilatory peptide ubiquitously found in tissues and organs, especially in cardiovascular tissues, kidneys, lungs, and endocrine glands [4]. Beside its vasoactive property, AM is known to have many pleiotropic effects, including mucosal healing and immunomodulation [5]. In particular, AM has been reported to be highly expressed in the gastrointestinal tract, with a high concentration of AM being found in the colon [6]. The beneficial effects of AM have been confirmed in an experimental rodent model of inflammatory bowel disease (IBD) [7–13]. In these experimental IBD models, AM was shown to suppress the inflammatory cytokines, stabilize the membrane, restore the vascular function, and accelerate ulcer reepithelialization and colon tissue regeneration [7–13]. More importantly, AM displayed considerable effects in seven patients with steroid-resistant UC in an exploratory clinical study [14]. These data suggested that AM might be a potent therapeutic candidate for UC because it acts via a novel mechanism that is not dependent on excessive immunosuppression and promotes mucosal regeneration. Additionally, as AM is an endogenous peptide, it would be expected to be safe for patients and could be added to existing drugs, such as steroids, immunosuppressants, and biologics. We have recently finished a phase 1 investigator-initiated clinical trial in healthy Japanese men and confirmed the safety and tolerability of AM [15]. After that, we have prepared a new AM formulation and planned a phase-2a investigator-initiated clinical trial for patients with intractable UC, due to the lack of support from industrial companies. In this multi-center, randomized, double-blind, placebo-controlled study, we evaluated the efficacy and safety of AM in Japanese patients with steroid-resistant UC. We also evaluated the dose-dependency of the response.

## Materials and methods

### Drug formulation

Briefly, AM is a peptide containing 52 amino acid residues with a ring structure formed by one intramolecular disulfide bond and an amidated structure formed by a C-terminal tyrosine [16]. We have produced a new lot of AM formulation for this phase 2 clinical trial utilizing the same manufacturing process according to the good manufacturing practice (GMP), as previously reported [15]. Briefly, the active pharmaceutical ingredient was chemically synthesized under GMP by the Peptide Institute (Osaka, Japan). Subsequently, the active pharmaceutical ingredient was dissolved in water containing d-mannitol and formulated as a freeze-dried material by Fuji Yakuhin (Toyama, Japan). Accordingly, a vial of AM for injection contained 500 μg AM and 50 mg d-mannitol. Likewise, a vial of placebo, which was indistinguishable from a vial of AM, contained 50 mg d-mannitol only. Vials were stored at 2–8 °C. Both the first and present lot of AM formulation have been subjected to a stability test conducted for 48 months by Fuji Yakuhin. Results confirmed the stability of our AM formulation for up to 48 months.

### Study design

This phase-2a, randomized, double-blind, multi-center, placebo-controlled study was conducted at 17 medical centers in Japan. After approval by the Pharmacological and Medical Device Agency (PMDA), ethical approval for this study was obtained from the Institutional Review Board of the University of Miyazaki and those of the other centers. This clinical trial was conducted in compliance with ethical principles based on the Declaration of Helsinki, good clinical practice (GCP) of the Japanese Ministerial Ordinance, and other related regulation requirements. The trial is registered in the JAPIC clinical trials information; Japic CTI- 205255 (200410115290).

### Subjects

Eligible patients were 18–75 years of age, had a definitive diagnosis of active UC, and were receiving at least a daily treatment of 30 mg or more of prednisolone for 7 days or more. Patients had to meet all criteria, namely, a Mayo score of 6 or more, a rectal bleeding subscore of at least 1, and Mayo endoscopy subscore (MES) of at least 2, at entry. Main exclusion criteria were as follows: patients with fulminant UC, patients with precancerous lesion in the colon, medication history of biologics within the last 3 months, patients with malignancy or any history of malignancy, and patients with active infection. We excluded patients with a systolic blood pressure under 90 mmHg and a pulse rate under 45 bpm for safety reasons due to the vasodilatory effect of AM. All patients provided written informed consent for all study-related procedures and were requested to be hospitalized for 2 weeks.

### Randomization and masking

Eligible patients were enrolled for the study by the principal investigator or a designee based on the above inclusion and exclusion criteria. Patients were randomized to one of four groups at a rate of 1:1:1:1 to receive placebo or 5, 10, or 15 ng/kg/min of AM. Randomization was performed by an independent contract research organization (CRO), CAC croit (Sapporo, Japan), using a block size of eight to attain a 1:1:1:1 ratio of randomization to placebo or three doses of AM. The same CRO prepared the medication (vials containing AM or placebo were indistinguishable) according to the randomization list. Randomization lists were kept under rigid control and opened after the completion of the study procedure and confirmation of data.

### Screening and evaluation

Patients were enrolled after confirmation of steroid-resistance defined as at least daily treatment with 30 mg or more of prednisolone. Screening tests including measurements of vital signs, blood and urine examinations, as well as a 12-lead electrocardiography (ECG), were conducted within 2 weeks prior to administration. Demographic data and disease characteristics including endoscopic disease activity of patients were also collected before randomization. Endoscopic subscores were assessed by on-site investigators and were not subjected to central review. Clinical disease activity was assessed by expert doctors according to the Mayo score [17] and Lichtiger index [18]. Stool samples were collected before administration of the investigational drug and after 2 weeks of treatment initiation. Measurements of fecal calprotectin and immunochemical blood tests (FITs) were performed by a commercially available laboratory testing service (BML, Tokyo, Japan) in compliance with GCP. Additionally, measurements of complement components including CH50, C1q, C3, and C4 and evaluation of the absolute number of hematopoietic stem cells in the peripheral blood were performed before and after 2, 4, and 8 weeks by other laboratory testing services (SRL medisearch, Tokyo, Japan and LSI medience, Tokyo, Japan, respectively). The absolute number of hematopoietic stem cells in the peripheral blood was measured as CD34-positive cells using CD45FITC/CD34PE, 7AAD kit (Becton Dickinson Immunocytometry System, USA) and flow cytometry.

### Interventions

After hospitalization, patients received a continuous infusion of the assigned drug for 8 h per day for a total of 14 days. After the final administration of the investigational drug, patients were subjected to safety examinations and then discharged. But, if necessary due to disease activity, continuous hospitalization was allowed. Patients were requested to make an office visit at 4 and 8 weeks. Evaluations of their Mayo scores including endoscopic examinations were done at 2 and 8 weeks, whereas evaluation of the Lichtiger index was done at 2, 4, and 8 weeks. The dose of concomitant steroid was maintained for 2 weeks after drug administration, and then, a reduction in the steroid dose was allowed depending on the disease condition. Changes to the doses of other drugs, such as aminosalicylic acid, were prohibited throughout the trial.

### Data assessment

All data were collected at each institute from March 2017 to April 2019. The primary endpoint was the change in the Mayo scores at 2 weeks. Secondary endpoints included the change in the Mayo scores at 8 weeks and the rate of clinical remission at 2 and 8 weeks, defined as a Mayo score 0; the response rate of the Mayo scores at 2 and 8 weeks, defined as an improvement of a 3-point or more decrease or at least a 30% decrease from baseline; the changes in MES at 2 and 8 weeks; the changes in the Lichtiger index at 2, 4, and 8 weeks; the changes in fecal calprotectin and FIT at 2 weeks; and finally, the changes in the given dose of steroid.

Safety evaluations including the evaluation of adverse events (AEs) and serious adverse events (SAEs) were conducted throughout the study. Vital signs, blood and urine tests, and a 12-lead ECG were performed at specified times after administration, with monitoring of ECG being assessed from the beginning of every test drug administration until 2 h after the administration.

Serum concentrations of AM were assessed at predose, 4 and 8 h, and 2 h after administration (10 h after the start of drug administration) on 1, 8, and 14 days of the administration. Blood sampling and the procedures for the measurement of the AM concentration have been previously described [15]. Measurements and data processing were carried by the Bozo Research Centre (Tsukuba, Japan). The evaluated pharmacokinetic (PK) parameters included the maximum measured plasma concentration (*C*_max_); the time to maximum measured plasma concentration (*T*_max_); and the cumulative area under the plasma concentration–time curve (AUC) from time 0 to time 10 h.

### Data collection and statistical analysis

All data, except for the PK parameters, were collected using an electronic data collection system (Viedoc) and analyzed by Intellim (Tokyo, Japan), an independent CRO. The primary full analysis set (FAS) included all patients who had completed 14 days of administration of the test drug. The population included in the safety analysis were all patients who received at least 1 day of administration of the test drug. Changes in Mayo scores, Lichtiger index, MES, fecal calprotectin, and FIT, as well as dose of steroid, were analyzed using unpaired *t* test and analysis of variance (ANOVA) followed by Fisher’s multiple comparison test. The clinical remission rate and response rate were analyzed using chi-square analysis. The significant level for each test was 5%. All analyses were performed using SAS version 9.3 (SAS Institute, Cary, NC, USA). The *C*_max_ and *T*_max_ values were obtained directly from the data, while the AUC was calculated using the Phoenix WinNonlin software 6·1 (Pharsight, CA, USA). All data are shown as mean ± standard deviation (SD).

## Results

### Randomization and clinical characteristics of patients at baseline

We obtained informed consent from 30 patients, and then two patients were excluded due to unfitness to the criteria. The remaining 28 patients were enrolled in this study, and 26 patients received the drug, but five patients dropped out within 14 days, and thus 21 patients completed the 14 days administration. Of the 21 patients, only 12 patients completed the 8 weeks follow-up. Main reasons for early drop-out were bad condition or not improved condition of the UC. The clinical characteristics of the 21 patients who completed the 14 days administration, adapted for FAS, are shown in Table 1. We did not observe any significant differences among the four groups except for the C-reactive protein (CRP) level. None of the patients received any biologics (infliximab or adalimumab) and immunosuppressants (cyclosporine or tacrolimus) for at least 3 months before their enrollment.

**Table 1:** Basal characteristics of the patients

Characteristics	Placebo	Adrenomedullin		
		5 ng/kg/min	10 ng/kg/min	15 ng/kg/min
Number of cases	6	4	5	6
Age (years)	48.0 ± 16.5	54.0 ± 16.1	38.2 ± 10.4	41.5 ± 19.8
Male/female	5/1	2/2	4/1	2/4
Body weight (kg)	56.3 ± 17.0	54.2 ± 7.6	69.1 ± 13.1	50.2 ± 10.2
Current smorking	5 (83%)	1 (25%)	2 (40%)	1 (17%)
Extent of the disease				
Pancolitis	5 (83%)	2 (50%)	3 (60%)	5 (83%)
Left-sided colitis	1 (17%)	2 (50%)	2 (40%)	1 (17%)
Mayo score	9.2 ± 1.5	9.5 ± 1.0	8.8 ± 1.1	9.8 ± 1.6
Lichitiger index	10.3 ± 2.4	10.8 ± 2.9	12.2 ± 0.4	12.0 ± 1.7
MES	2.5 ± 0.5	2.3 ± 0.5	2.2 ± 0.4	2.5 ± 0.5
FIT (ng/ml)	9615 ± 10.106	2358 ± 1989	1516 ± 2655	15020 ± 21896
Fecal calprotectin (mg/kg)	13639 ± 7068	15416 ± 10337	12780 ± 14307	17723 ± 15284
CRP (mg/dl)	0.66 ± 0.62	0.08 ± 0.07*	0.99 ± 1.13	2.27 ± 1.65
adrenomedullin (pg/ml)	9.6 ± 3.2	10.1 ± 0.6	10.6 ± 3.2	15.5 ± 3.9
Dose of steroid (mg)	29.2 ± 17.7	20.0 ± 11.5	24.0 ± 20.7	31.7 ± 15.1

*MES* Mayo endoscopy sub-score, *FIT* fecal immunochemical blood test, *CRP* C-reactive protein

*P = 0.023, compared to 15 ng/kg/min

### Clinical efficacy

**Fig. 1:**
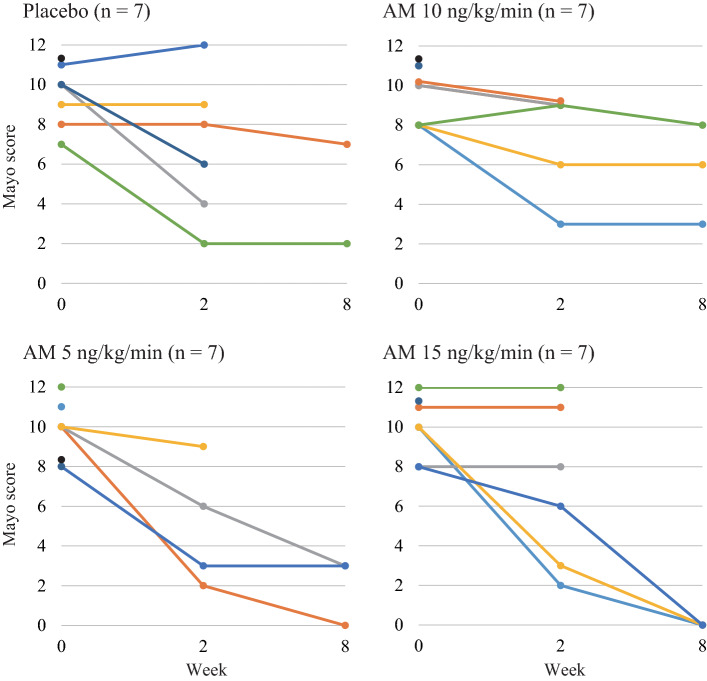
Time course of each Mayo score in all patients

**Fig. 2:**
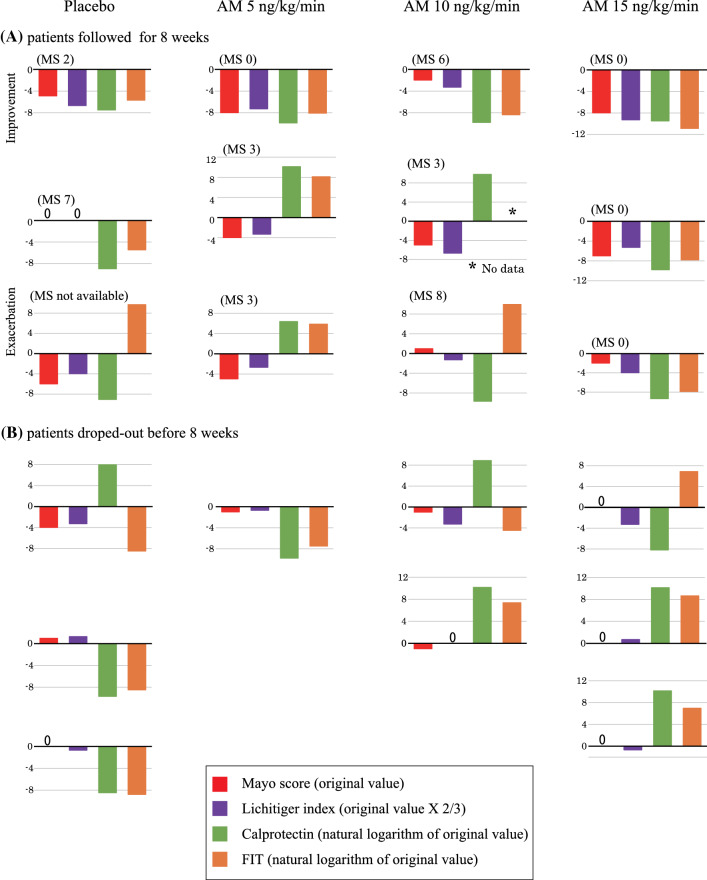
Changes in clinical indexes (Mayo score and Lichtiger index) and fecal markers (calprotectin and immunochemical blood test) of each patient at 2 weeks. Changes in markers that showed exacerbation or improvement of the disease are grouped together. Negative values mean improvement, whereas positive values mean exacerbation. Mayo score (MS) 0 or more indicate the MS at 8 weeks, whereas not listed means dropped out patients before 8 weeks

We did not observe any differences among the four groups for the primary endpoint, the changes in Mayo scores at 2 weeks (Table 2). No patient was noted to achieve complete remission at 2 weeks. Although the tracking rate was insufficient, the Mayo score at 8 weeks was shown to be significantly decreased only in the group of patients receiving high-dose AM (15 ng/kg/min) compared with the placebo group (− 9.3 ± 1.2 vs. − 3.0 ± 2.8, *P* = 0.035). However, it should be mentioned that one patient in the placebo group refused endoscopic examination, and hence, the Mayo score was not assessed in this patient. More importantly, all patients in the high AM dose group were demonstrated to achieve complete remission at 8 weeks, and this was statistically significant compared with the placebo group (Table 2). The time course of the Mayo score in each patient is illustrated in Fig. 1. All patients with a serious condition (Mayo score ≥ 11) at basal status showed no improvement and were dropped out within 2 weeks. One patient in the low-dose AM group and three patients in the high-dose AM group reached complete remission by 8 weeks. We observed a similar tendency in the Lichtiger index (Table 3), where the decrease in the Lichtiger index reached marginal range (*P* = 0.075) only in the high-dose AM group at 8 weeks. No differences were noted among the four groups for MES (see Supplementary Table 1). The doses of steroids in all patients were reduced after 2 weeks administration of the investigated drug. The doses of steroid at 8 weeks for placebo, AM 5 ng/kg/min, AM 10 ng/kg/min, and AM 15 ng/kg/min groups were 11.3 ± 6.4 (*n* = 3), 5.0 ± 8.7 (*n* = 3), 10.0 ± 10.0 (*n* = 3), and 16.7 ± 12.6 mg (*n* = 3), respectively. The changes in steroid doses between 0 and 8 weeks for each group were − 8.7 ± 7.1, − 18.3 ± 10.4, − 6.7 ± 11.5, and − 23.3 ± 7.6 mg, respectively. However, we did not identify any statistical differences among the four groups in the changes in the steroid dose at 4 and 8 weeks. We also did not observe any differences in fecal calprotectin and FIT among the four groups at 2 weeks. The changes in fecal calprotectin and FIT in each patient are depicted in Fig. 2, along with the changes in clinical indexes. Finally, no meaningful changes were observed in the complement components and absolute number of hematopoietic stem cells in peripheral blood in all groups at all measured time-points (see Supplementary Table 2).

**Table 2:** Changes of Mayo score

	Placebo		Adrenomedullin					
			5 ng/kg/min		10 ng/kg/min		15 ng/kg/min	
	*n*	Mean ± SD	*n*	Mean ± SD	*n*	Mean ± SD	*n*	Mean ± SD
Mayo score								
Baseline	6	9.2 ± 1.5	4	9.5 ± 1.0	5	8.8 ± 1.1	6	9.8 ± 1.6
2 weeks	6	6.8 ± 3.6	4	5.0 ± 3.2	5	7.2 ± 2.7	6	7.0 ± 4.1
8 weeks	2	4.5 ± 3.5	3	2.0 ± 1.7	3	5.7 ± 2.5	3	0.0 ± 0.0
Change of Mayo score								
2 weeks	6	− 2.3 ± 3.0	4	− 4.5 ± 2.9	5	− 1.6 ± 2.2	6	− 2.8 ± 3.0
8 weeks	2	− 3.0 ± 2.8	3	− 7.3 ± 2.5	3	− 2.3 ± 2.5	3	− 9.3 ± 1.2
*P* value (vs. placebo)								
2 weeks	6	–	4	0.29	5	0.66	6	0.80
8 weeks	2	–	3	0.17	3	0.80	3	0.035
Response (response/non-response, %)								
2 weeks	6	3/3 (50%)	4	3/1 (75%)	5	1/4 (20%)	6	2/4 (33%)
8 weeks	2	1/1 (50%)	3	3/0 (100%)	3	1/2 (33%)	3	3/0 (100%)
*P* value (vs. placebo)								
2 weeks	6	–	4	0.43	5	0.30	6	0.56
8 weeks	2	–	3	0.17	3	0.71	3	0.17
Remission (Mayo score 0/others, %)								
2 weeks	6	0/6 (0%)	4	0/4 (0%)	5	0/5 (0%)	6	0/6 (0%)
8 weeks	2	0/2 (0%)	3	1/2 (33%)	3	0/3 (0%)	3	3/0 (100%)
*P* value (vs. placebo)								
2 weeks	6	–	4	N/A	5	N/A	6	N/A
8 weeks	2	–	3	0.36	3	N/A	3	0.025

**Table 3:** Changes of Lichitiger index

	Placebo		Adrenomedullin					
			5 ng/kg/min		10 ng/kg/min		15 ng/kg/min	
	*n*	Mean ± SD	*n*	Mean ± SD	*n*	Mean ± SD	*n*	Mean ± SD
Lichitiger index								
Baseline	6	10.3 ± 2.4	4	10.8 ± 2.9	5	12.2 ± 0.4	6	12.0 ± 1.7
2 weeks	6	7.0 ± 5.8	4	5.5 ± 5.6	5	7.8 ± 3.8	6	6.5 ± 4.4
4 weeks	4	3.5 ± 3.7	3	2.0 ± 1.7	3	4.7 ± 4.7	3	1.3 ± 1.5
8 weeks	3	2.3 ± 4.0	3	1.3 ± 1.2	3	5.7 ± 4.5	3	0.3 ± 0.6
Change of Lichitiger index								
2 weeks	6	− 3.3 ± 4.5	4	− 5.3 ± 4.2	5	− 4.4 ± 3.8	6	− 5.5 ± 5.3
4 weeks	4	− 5.8 ± 4.2	3	− 7.7 ± 3.5	3	− 7.3 ± 4.7	3	− 11.7 ± 3.2
8 weeks	3	− 6.0 ± 4.6	3	− 8.3 ± 3.1	3	− 6.3 ± 4.5	3	− 12.7 ± 1.5
*P* value (vs. placebo)								
2 weeks	6	–	4	0.52	5	0.68	6	0.46
4 weeks	4	–	3	0.55	3	0.66	3	0.10
8 weeks	3	–	3	0.50	3	0.93	3	0.075

### Plasma concentration of adrenomedullin

**Fig. 3:**
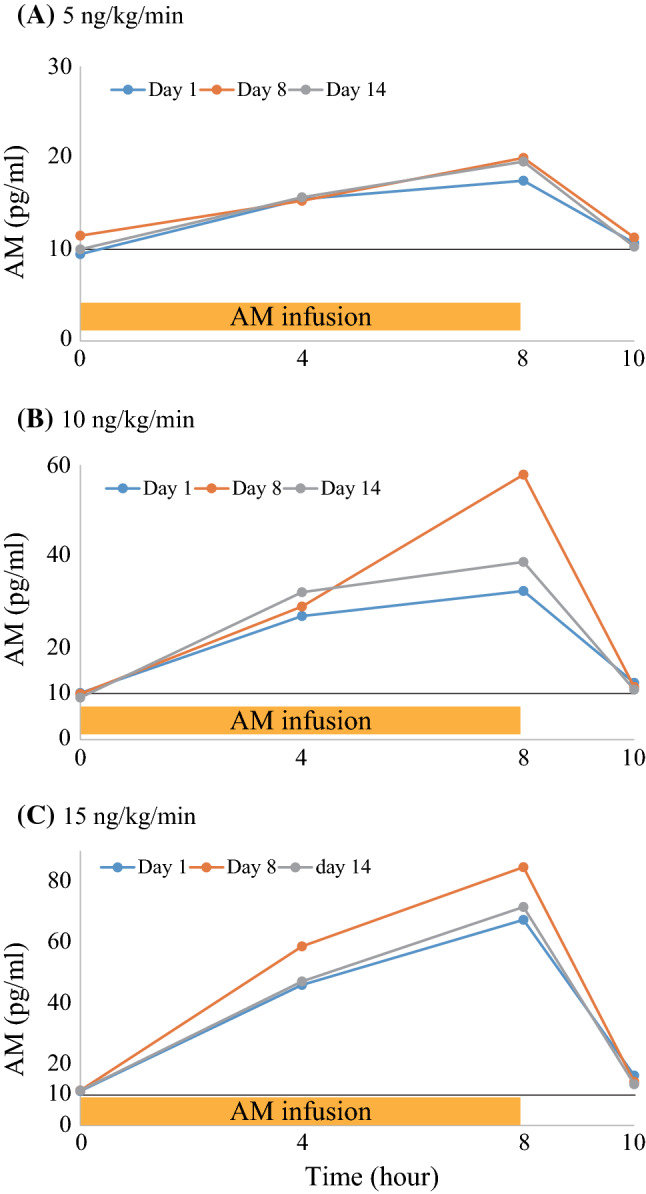
Mean plasma concentration–time profiles of adrenomedullin at 1, 8, and 14 days of repeated infusion

Plasma concentrations were dose-dependently increased by AM infusion with good reproducibility after repeated administration (Fig. 3). The increased plasma concentration of AM mostly returned to basal levels within 2 h after the termination of the AM infusion. We did not observe any progressive increase in the plasma concentration of AM after repeated administrations, which is a concern in multiple-dose administration studies in a phase 1 trial [15]. The AUC_0-10 h_ after 14 days of the three groups, namely 5, 10, and 15 ng/kg/min groups, was shown to be 152 ± 34, 281 ± 39, and 440 ± 191 h*pg/mL, respectively. The AUC_0-10 h_ after 14 days of the high-dose AM group (15 ng/kg/min) was significantly higher than that of the other groups (*P* < 0.05).

### Safety assessments

Out of the 26 patients who received investigational drugs, five were dropped out within 14 days; one patient each in the placebo, 5 ng/kg/min, and 15 ng/kg/min groups and two patients in the 10 ng/kg/min group. Only one patient in the 10 ng/kg/min group terminated the administration because of headaches and a decrease in blood pressure, which were caused due to the vasodilatory effect of AM. However, these AEs were observed to quickly disappear after termination of drug administration. The reported AEs are summarized in Table 4. Symptoms related to the vasodilatory effect of AM, such as headaches, palpitations, decreases in blood pressure, and flushes, were more frequently observed in the AM-treated groups than the placebo group. However, these symptoms were mild and tolerable without any treatments, except for the one patient mentioned above. We did not observe any statistically significant changes in blood pressure and heart rate in all groups throughout the drug administration period (see Supplementary Fig. 1). An SAE reported to have occurred in one patient in the placebo group was that of infectious pneumonia (*Pneumococcus* + *Pneumocystis*) and related adrenal insufficiency 1 month after the end of the placebo administration. This patient recovered after appropriate treatment, and this SAE was judged by the investigators to not be related to the study drug. However, we did observe gastrointestinal disorders or increases in the levels of transaminase, which were probably related to UC, in both the placebo and the AM groups. No deaths occurred during this trial.

**Table 4:** Summary of reported adverse events

	Placebo [7]	Adrenomedullin		
		5 ng/kg/min [5]	10 ng/kg/min [7]	15 ng/kg/min [7]
Any adverse events	4 (57%)	4 (80%)	6 (86%)	6 (86%)
Severe adverse events	0	0	0	0
Serious adverse events	1 (14%)	0	0	0
Deaths	0	0	0	0
Major adverse events				
Nervous system disorders				
Head disconfort	0	0	1 (14%)	2 (29%)
Headache	1 (14%)	3 (60%)	4 (57%)	4 (57%)
Cardiovascular disorders				
Palpitation	0	0	0	1 (14%)
Flushing	0	0	0	2 (29%)
Decrease of blood pressure	0	0	1 (14%)	1 (14%)
Others				
Gastrointestinal disorders	0	2 (40%)	0	0
Increase of transaminase	2 (29%)	0	0	1 (14%)
Muscle pain, back pain	2 (29%)	0	0	0
Rash	0	0	0	1 (14%)

## Discussion

This is the first randomized, placebo-controlled, phase-2a trial of AM in Japanese patients with steroid-resistant UC. Including the primary endpoint, none of the selected parameters showed any improvements following treatment with AM compared with treatment with placebo at 2 weeks. However, statistically significant remission was achieved by high-dose AM (15 ng/kg/min) at 8 weeks. Unfortunately, the low and medium doses of AM (5 and 10 ng/kg/min) did not show any clear improvement in patients even at 8 weeks, so a dose–response relationship was not suggested. Except for one patient in the medium-dose AM group, all patients exhibited high tolerance for the treatment with AM, with no SAE occurring following administration of AM.

AM is thought to work as an endogenous counter-factor for UC, and thus, increases in AM production in UC patients were suggested. We found that the basal plasma concentration of AM was higher in patients with UC than that in healthy volunteers in the phase 1 single-dose study (11.4 ± 7.2 pg/mL (*n* = 20) vs. 7.2 ± 1.4 pg/mL (*n* = 23); *P* < 0.0001) [15]. Of note, in one UC patient in the present study, this value was found to be below the lower limit of quantification (< 5 pg/mL). At administration, 14 of 21 patients received infusion therapy and/or enteral nutrition, and normal diets were restricted (five in the placebo group, three in the low-dose group, two in the middle-dose group, and four in the high-dose group). Plasma concentrations of AM in patients with and without a restricted diet were 12.5 ± 4.2 (*n* = 13) and 10.6 ± 2.9 pg/mL (*n* = 7), respectively (*P* = 0.25).

A high rate of the placebo effect has been reported in a previous trial for UC [1–3]. High response rates in the placebo group have occasionally obstructed the successful accomplishment of clinical trials for active UC [19]. In this trial, we were concerned about the major impact from the placebo effect because attending patients received hospitalization and bed rest for 2 weeks. Figure 2 illustrates the changes in clinical indexes and fecal markers of each patient at 2 weeks. Interestingly, patients who achieved total remission at 8 weeks (indicated as MS 0) showed clear improvements in all clinical and fecal markers. These patients probably reflected the real effect of AM. In contrast, we observed a dissociation between clinical indexes and fecal markers in resting patients. A relatively good response of all markers was observed even in the placebo group (indicated as MS 2). Additionally, in the placebo group, we identified small improvements in clinical indexes with inconsistent changes in fecal markers or almost no changes in clinical indexes with remarkable improvements in fecal markers. These results suggested that 2 weeks of bed rest more or less influenced all patients, and this might be the reason why we observed nonsignificant or similar changes in markers at 2 weeks. All patients were discharged after treatment for 2 weeks, so we could not detect a continued improvement in the placebo group from 2 to 8 weeks (Fig. 1). On the contrary, we observed a progressive improvement in the high-dose AM group in the same period, indicating that this must have been the real effect of AM. Similar improvements might have occurred in the low-dose group, but not in the medium-dose AM group (Fig. 1). We previously reported a significant improvement in steroid-resistant UC with administration of 9 ng/kg/min of AM, a dose similar to the medium dose of AM in this trial [14]. Although the details were unclear, one possible reason for this discrepancy might have been the different methods of preparing the AM formulation. Previously, based on our experience, we used a 1.4-fold dose of the bulk AM powder to compensate for internal water contamination in the bulk powder, so the real dose of the AM formulation might have been higher than the attributed 9 ng/kg/min.

The importance of early mucosal healing has recently attracted a lot of attention because of the associated sustained clinical remission and reduced operation rate in active UC [20–21]. The rates of mucosal healing in the short-term have been reported to be 43.1–60.7% in randomized control trials (RCTs) using anti-TNFα biologics against moderate-to-severe UC [1–2, 22–24]. A high dose of AM was shown to achieve comparable rates of mucosal healing, namely 50% (three of six patients who received a 2 weeks administration of AM) in this trial (Fig. 1). Additionally, mucosal healing in previous studies was defined as an MES of 0–1 [1–2, 22–24], but all successfully treated patients receiving AM achieved an MES of 0 (see Supplementary Table 1). As AM is known to be an endogenous peptide, it would probably be safe for patients and could be added to the list of existing drugs, such as steroids, immunosuppressants, and biologics without fear for excessive immunosuppression. Indeed, we did not observe any occurrence of critical or unexpected AEs in this trial following administration of AM (Table 4). It is expected that AM could serve as an alternative agent for the induction of mucosal healing with minimum risk in patients with UC. In contrast, AM could not improve the disease state in the more severe patients with a high Mayo score of 11–12 (Fig. 1). Therefore, AM might not exhibit an immediate effect introduced by tacrolimus in the more severe or fulminant UC [25]. As AM is suggested to promote mucosal regeneration without strong immunosuppression, some hold time might be needed for mucosal healing.

It was previously reported that AM and its binding protein, complement factor H, downregulated the levels of inflammatory cytokines and attenuated tissue injury in gut ischemia and reperfusion injury [26, 27]. Additionally, AM and complement factor H have been shown to enhance the cleavage of C3b via factor I [28]. So, we speculated that administration of AM might affect the complement factors and contribute to tissue repairing in patients with UC. Unfortunately, we did not detect any significant changes in complement factors following administration of AM (Supplement Table 2). We also did not observe any short-term changes in the complement factors during administration of AM (data not shown). It has been reported that AM might promote angiogenesis through circulating bone-marrow derived cells [29]. Unfortunately, we did not observe any significant changes in the absolute number of hematopoietic stem cells in the peripheral blood (Supplement Table 2).

This study had several limitations. First, the number of patients was small even though this was an early phase 2 trial. In addition, almost half of the patients dropped out after 2 weeks of treatment. This might have interfered with the detection of the true therapeutic effect of AM. Additionally, AM was not effective in the more severe cases of patients, and a dose–response relationship was not shown. Appropriate targeting of patients and a different dose setting will be needed in a following trial. Second, the endoscopic subscores were not subjected to central review, so interobserver variations might have been unavoidable. Fortunately, these variations might have been negligible for the complete remission at 8 weeks. Finally, the placebo effect associated with the 2 weeks bed rest might have masked the effects of AM at 2 weeks.

In conclusion, despite the limited number of patients in this double-blind randomized trial, we observed the successful complete remission at 8 weeks in patients with steroid-resistant UC receiving a high dose of AM. Hence, AM could serve as candidate potent therapeutic agent for complete remission in refractory UC.

## Electronic supplementary material

Below is the link to the electronic supplementary material.

Supplementary file1 Time course of systolic blood pressure and pulse rate of patients in each group. Data are arithmetic mean of the repeated administration of each drug (day 1 to day 14). (PDF 412 kb)

Supplementary file1 (PDF 24 kb)

Supplementary file1 (PDF 107 kb)

## References

[1] Rutgeerts P, Sandborn WJ, Feagan BG (2005). Infliximab for induction and maintenance therapy for ulcerative colitis. N Engl J Med.

[2] Reinisch W, Sandborn WJ, Hommes DW (2011). Adalimumab for induction of clinical remission in moderately to severely active ulcerative colitis: results of a randomized controlled trial. Gut.

[3] Feagan BG, Rutgeerts P, Sands BE (2013). Vedolizumab as induction and maintenance therapy for ulcerative colitis. N Engl J Med.

[4] Eto T, Kato J, Kitamura K (2002). Regulation of production and secretion of adrenomedullin in the cardiovascular system. Regl Pept.

[5] Cheung BM, Tang F (2012). Adrenomedullin: exciting new horizons. Recent Pat Endocr Metab Immune Drug Discov.

[6] Sakata J, Asada Y, Shimokubo T (1998). Adrenomedullin in the gastrointestinal tract. Distribution and gene expression in rat and augmented gastric adrenomedullin after fasting. J Gastroenterol.

[7] Ashizuka S, Ishikawa N, Kato J (2005). Effect of adrenomedullin administration on acetic acid-induced colitis in rats. Peptides.

[8] Gonzales-Rey E, Fernandez-Martin A, Chorny A (2006). Therapeutic effect of urocortin and adrenomedullin in a murine model of Crohn’s disease. Gut.

[9] Talero E, Sánchez-Fidalgo S, de la Lastra CA (2008). Acute and chronic responses associated with adrenomedullin administration in experimental colitis. Peptides.

[10] Ashizuka S, Inagaki-Ohara K, Kuwasako K (2009). Adrenomedullin treatment reduces intestinal inflammation and maintains epithelial barrier function in mice administered dextran sulphate sodium. Microbiol Immunol.

[11] Talero E, Alvarez de Sotomayor M, Sánchez-Fidalgo S (2011). Vascular contribution of adrenomedullin to microcirculatory improvement in experimental colitis. Eur J Pharmacol.

[12] Hayashi Y, Narumi K, Tsuji S (2011). Impact of adrenomedullin on dextran sulfate sodium-induced inflammatory colitis in mice: insights from in vitro and in vivo experimental studies. Int J Colorectal Dis.

[13] Kinoshita Y, Arita S, Murazoe H (2019). Subcutaneously administered adrenomedullin exerts a potent therapeutic effect in a murine model of ulcerative colitis. Hum Cell.

[14] Ashizuka S, Inatsu H, Kita T (2016). Adrenomedullin therapy in patients with refractory ulcerative colitis: a case series. Dig Dis Sci.

[15] Kita T, Kaji Y, Kitamura K (2020). Safety, tolerability, and pharmacokinetics of adrenomedullin in healthy males: a randomized, double-blind, phase 1 clinical trial. Drug Des Dev Ther.

[16] Kitamura K, Kangawa K, Kawamoto M (1993). Adrenomedullin: a novel hypotensive peptide isolated from human pheochromocytoma. Biochem Biophys Res Commun.

[17] Schroeder KW, Tremaine WJ, Ilstrup DM (1987). Coated oral 5-aminosalicylic acid therapy for mildly to moderately active ulcerative colitis. A randomized study. N Engl J Med.

[18] Lichtiger S, Present DH, Kornbluth A (1994). Cyclosporine in severe ulcerative colitis refractory to steroid therapy. N Engl J Med.

[19] Su C, Lewis JD, Goldberg B, Brensinger C (2007). A meta-analysis of the placebo rates of remission and response in clinical trials of active ulcerative colitis. Gastroenterology.

[20] Colombel JF, Rutgeerts P, Reinisch W (2011). Early mucosal healing with infliximab is associated with improved long-term clinical outcomes in ulcerative colitis. Gastroenterology.

[21] Sandborn WJ, Rutgeerts P, Feagan BG (2009). Colectomy rate comparison after treatment of ulcerative colitis with placebo or infliximab. Gastroenterology.

[22] Sandborn WJ, van Assche G, Reinisch W (2012). Adalimumab induces and maintains clinical remission in patients with moderate-to-severe ulcerative colitis. Gastroenterology.

[23] Panaccione R, Ghosh S, Middleton S (2014). Combination therapy with infliximab and azathioprine is superior to monotherapy with either agent in ulcerative colitis. Gastroenterology.

[24] Colombel JF, Sandborn WJ, Ghosh S (2014). Four-year maintenance treatment with adalimumab in patients with moderately to severely active ulcerative colitis: data from ULTRA 1, 2, and 3. Am J Gastroenterol.

[25] Ogata H, Kato J, Hirai F (2012). Double-blind, placebo-controlled trial of oral tacrolimus (FK506) in the management of hospitalized patients with steroid-refractory ulcerative colitis. Inflamm Bowel Dis.

[26] Carrizo GJ, Wu R, Cui X (2007). Adrenomedullin and adrenomedullin-binding protein-1 downregulate inflammatory cytokines and attenuate tissue injury after gut ischemia-reperfusion. Surgery.

[27] Zhang F, Wu R, Zhou M (2009). Human adrenomedullin combined with human adrenomedullin binding protein-1 is protective in gut ischemia and reperfusion injury in the rat. Regul Pept.

[28] Pío R, Martínez A, Unsworth EJ (2001). Complement factor H is a serum-binding protein for adrenomedullin, and the resulting complex modulates the bioactivities of both partners. J Biol Chem.

[29] Abe M, Sata M, Suzuki E (2006). Effects of adrenomedullin on acute ischaemia-induced collateral development and mobilization of bone-marrow-derived cells. Clin Sci (Lond).

