# Supplementary figures and images for: Adrenomedullin for steroid-resistant ulcerative colitis: a randomized, double-blind, placebo-controlled phase-2a clinical trial
Source: J Gastroenterol. 2020 Nov 2;56(2):147–57. doi: 10.1007/s00535-020-01741-4 (PMC7862507; doi:10.1007/s00535-020-01741-4)

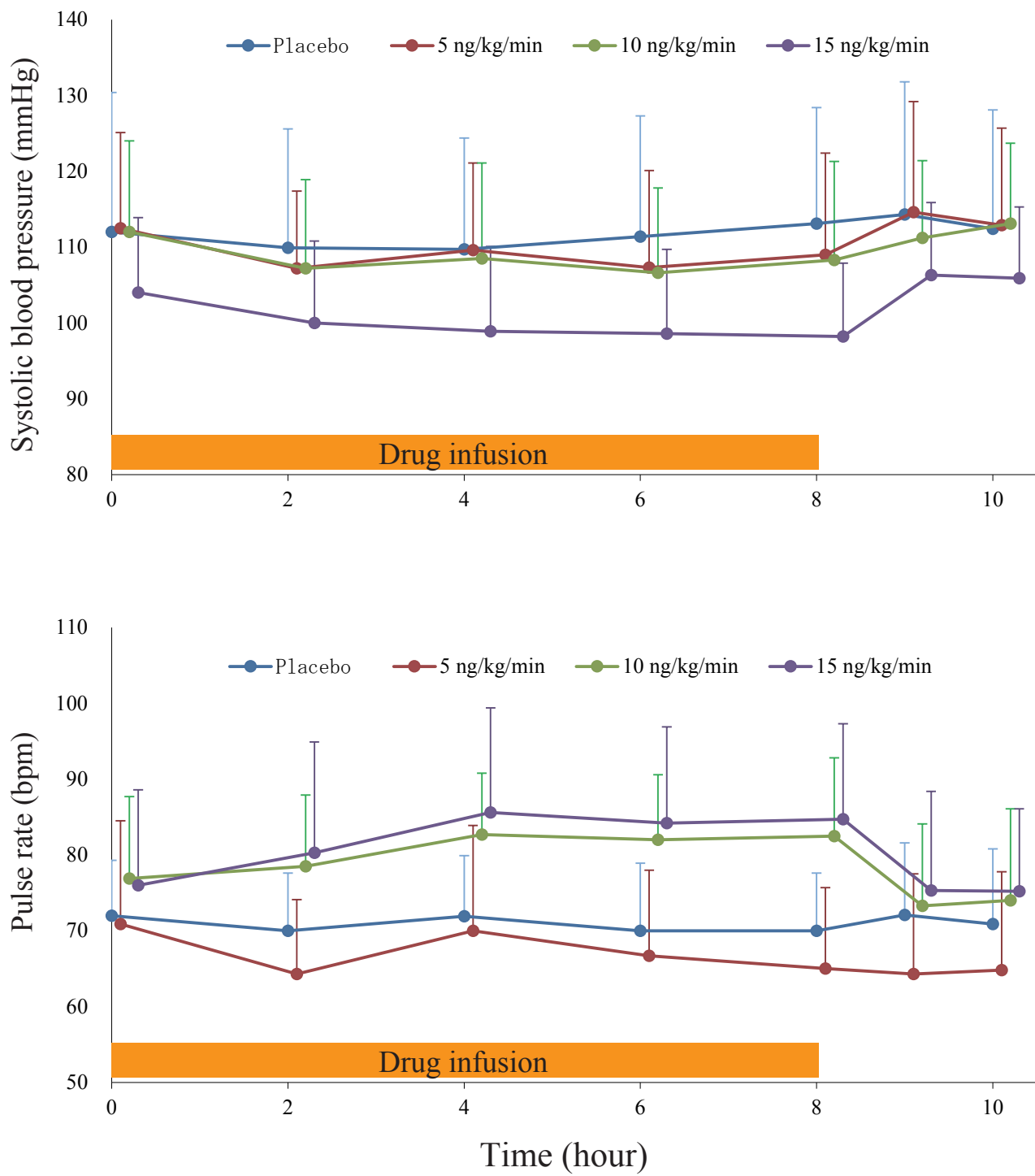

Supple. Figure 1

Supplement: Supplementary file 1 — Supplementary file1 Time course of systolic blood pressure and pulse rate of patients in each group. Data are arithmetic mean of the repeated administration of each drug (day 1 to day 14). (PDF 412 kb) [file 535_2020_1741_MOESM1_ESM.pdf]
