# Supplementary material for: Adrenomedullin for steroid-resistant ulcerative colitis: a randomized, double-blind, placebo-controlled phase-2a clinical trial
Source: J Gastroenterol. 2020 Nov 2;56(2):147–57. doi: 10.1007/s00535-020-01741-4 (PMC7862507; doi:10.1007/s00535-020-01741-4)
Supplement: Supplementary file 2 — Supplementary file1 (PDF 24 kb) [file 535_2020_1741_MOESM2_ESM.pdf]

Suppl. Table 1. Changes of MES

	Placebo		Adrenomedullin					
			5 ng/kg/min		10 ng/kg/min		15 ng/kg/min	
	n	Mean ± SD	n	Mean ± SD	n	Mean ± SD	n	Mean ± SD
MES								
baseline	6	2.5 ± 0.5	4	2.3 ± 0.5	5	2.2 ± 0.4	6	2.5 ± 0.5
2 week	6	2.0 ± 0.6	4	1.5 ± 0.6	5	2.2 ± 0.8	6	2.3 ± 1.0
8 week	2	2.0 ± 1.4	3	0.7 ± 0.6	3	2.0 ± 0.0	3	0.0 ± 0.0
Change of MES								
2 week	6	-0.5 ± 0.8	4	-0.8 ± 1.0	5	0.0 ± 0.7	6	-0.2 ± 0.8
8 week	2	-0.5 ± 2.1	3	-1.7 ± 1.2	3	0.0 ± 0.0	3	-2.0 ± 0.0
P value (vs. placebo)								
2 week	6	—	4	0.67	5	0.32	6	0.49
8 week	2	—	3	0.47	3	0.69	3	0.27

MES: Mayo endoscopic sub-score
