# Supplementary material for: Adrenomedullin for steroid-resistant ulcerative colitis: a randomized, double-blind, placebo-controlled phase-2a clinical trial
Source: J Gastroenterol. 2020 Nov 2;56(2):147–57. doi: 10.1007/s00535-020-01741-4 (PMC7862507; doi:10.1007/s00535-020-01741-4)
Supplement: Supplementary file 3 — Supplementary file1 (PDF 107 kb) [file 535_2020_1741_MOESM3_ESM.pdf]

Suppl. Table 2. Hematopoietic stem sells and complement components

	Placebo (n = 3)				Adrenor					
					5 ng/kg/min (n = 3)				10 ng/kg/n	
	Pre	2 W	4 W	8 W	Pre	2 W	4 W	8 W	Pre	2 W
HPSC (cells/μL)	5.0 ± 6.9	4.5 ± 5.6	5.8 ± 8.0	5.7 ± 8.1	0.6 ± 0.5	0.5 ± 0.5	0.7 ± 0.4	0.8 ± 0.4	0.7 ± 0.3	0.8 ± 0.2
CH50 (U/mL)	41.8 ± 6.1	45.6 ± 12.3	46.2 ± 10.8	45.2 ± 14.9	42.7 ± 2.2	42.6 ± 1.7	37.0 ± 2.7	43.7 ± 4.1	44.1 ± 0.9	44.2 ± 9.8
C1q (mg/dL)	8.3 ± 0.9	8.4 ± 1.2	11.3 ± 5.6	9.0 ± 0.6	8.6 ± 2.2	7.9 ± 1.0	7.7 ± 1.1	8.0 ± 0.6	10.4 ± 2.8	9.0 ± 2.5
C3 (mg/dL)	120 ± 13	128 ± 15	130 ± 3	140 ± 16	114 ± 17	110 ± 27	95 ± 13	121 ± 8	131 ± 9	126 ± 27
C4 (mg/dL)	23 ± 3	23 ± 3	23 ± 3	25 ± 6	35 ± 7	34 ± 4	27 ± 5	35 ± 13	30 ± 9	32 ± 11

HPSC: absolute number of hematopoietic stem cells in peripheral blood

nedullin					
nin (n = 3)		15 ng/kg/min (n = 3)			
4 W	8 W	Pre	2 W	4 W	8 W
0.9 ± 0.3	0.7 ± 0.3	1.9 ± 1.8	5.5 ± 6.7	2.3 ± 2.3	10.0 ± 13.9
42.4 ± 3.0	46.2 ± 0.8	53.7 ± 4.0	54.8 ± 10.4	47.4 ± 8.3	43.5 ± 6.9
10.2 ± 2.8	10.7 ± 2.2	9.5 ± 1.7	8.7 ± 2.4	11.1 ± 3.4	8.3 ± 1.6
120 ± 11	126 ± 8	150 ± 17	149 ± 6	128 ± 55	118 ± 39
27 ± 12	28 ± 7	35 ± 11	35 ± 11	23 ± 1	22 ± 2
